# Supplementary material for: Chromothripsis during telomere crisis is independent of NHEJ, and consistent with a replicative origin
Source: Genome Res. 2019 May;29(5):737–49. doi: 10.1101/gr.240705.118 (PMC6499312; doi:10.1101/gr.240705.118)
Supplement: Supplemental Material [file supp_gr.240705.118_Supplemental_file_1.zip › contigs/annotated_contigs/DB53/contig.2.DB53_length_311_mean_cov_3.0578778135.docx]

**DB53_length_311_mean_cov_3.0578778135**

```
AACATCAGCACTGATCTCAACCCCATCCCCACATCTGTAGAAGTTTCAAGATCACCAGGTGGCATAATAACAACA|T|CAAGTAATCTG
 >chr7:154886651-154886727 - E=2e-34                                        >chr1:16107
CCCGCCTTGGCCTACCAAACTGCTGGAATTACAGGCATGAGCCACCATGCCAGGCCTGTCCAAAGAGATTTTATATTGGTACTATTTAT
7220-161077390 - E=4e-91
TACAGAAAAATTGGAAACAACCTAAATGTTCTAACGCAGAGGATTAGTTAAGCTTTTTTTTTTTTTTTT|ATTTATTTTATTGTTTTAT

GTTCTTTTTTGGATTGTTGTTGTTTGTGGTTTTCTTTTTTCATTGTT
```
